# Dysregulated heme oxygenase-1^low^ M2-like macrophages augment lupus nephritis via Bach1 induced by type I interferons

**DOI:** 10.1186/s13075-018-1568-1

**Published:** 2018-04-10

**Authors:** Daiga Kishimoto, Yohei Kirino, Maasa Tamura, Mitsuhiro Takeno, Yosuke Kunishita, Kaoru Takase-Minegishi, Hiroto Nakano, Ikuma Kato, Kiyotaka Nagahama, Ryusuke Yoshimi, Kazuhiko Igarashi, Ichiro Aoki, Hideaki Nakajima

**Affiliations:** 1 Department of Stem Cell and Immune Regulation, Yokohama City University Graduate School of Medicine, 3-9 Fukuura, Kanazawa-Ku, Yokohama, 236-0004 Japan; 2 Department of Allergy and Rheumatology, Nippon Medical School Graduate School of Medicine, Tokyo, Japan; 3 Department of Molecular Pathology, Yokohama City University Graduate School of Medicine, Yokohama, Japan; 4 Department of Pathology, Kyorin University School of Medicine, Tokyo, Japan; 5 Department of Biochemistry, Tohoku University School of Medicine, Sendai, Japan; 6 Center for Regulatory Epigenome and Diseases, Tohoku University School of Medicine, Sendai, Japan

**Keywords:** Lupus nephritis, Heme oxygenase 1, Bach1, Type I interferons, Macrophage polarization

## Abstract

**Background:**

Innate immunity including macrophages (Mϕ) in lupus nephritis (LN) has been gaining attention, but roles of Mϕ in LN remain uncertain.

**Methods:**

Immunohistochemical staining was performed to determine CD68, CD163, heme oxygenase (HO)-1 (a stress-inducible heme-degrading enzyme with anti-inflammatory property), pSTAT1, and CMAF-expressing Mϕ in the glomeruli of patients with LN. Effects of type I interferons on the expression levels of CD163, HO-1, BTB and CNC homology 1 (Bach1; a transcriptional HO-1 repressor), interleukin (IL)-6, and IL-10 by human M2-like Mϕ, which were differentiated *in vitro* from peripheral monocytes with macrophage colony-stimulating factor, were assessed by RT-PCR and immunocytostaining. Clinical manifestations, anti-double-stranded DNA (anti-dsDNA), and local HO-1 expression were compared in Bach1-deficient and wild-type MRL/*lpr* mice.

**Results:**

The number of glomerular M2-like Mϕ correlated with the amounts of proteinuria in patients with LN. Unlike monocyte-derived M2-like Mϕ, HO-1 expression was defective in the majority of glomerular M2-like Mϕ of patients with LN. Stimulation of human M2-like Mϕ with type I interferons led to reduced HO-1 expression and increased Bach1 and IL-6 expression. Bach1-deficient MRL/*lpr* mice exhibited increased HO-1 expression in kidneys, prolonged survival, reduced urine proteins, and serum blood urea nitrogen levels, but serum anti-dsDNA antibody levels were comparable. Increased expression of CD163 and HO-1 was found in peritoneal Mϕ from Bach1-deficient MRL/*lpr* mice.

**Conclusions:**

Our data suggest that dysregulated M2-like Mϕ play a proinflammatory role in LN. Bach1 is a potential therapeutic target that could restore the anti-inflammatory property of M2 Mϕ.

**Electronic supplementary material:**

The online version of this article (10.1186/s13075-018-1568-1) contains supplementary material, which is available to authorized users.

## Background

Systemic lupus erythematosus (SLE) is an autoimmune disease with a broad spectrum of clinical presentations [1]. Lupus nephritis (LN) occurs in approximately 25–50% of patients with SLE and remains one of the leading causes of morbidity [2]. New immunosuppressive therapies such as mycophenolate mofetil have been improving disease outcomes in patients with SLE, but some patients are refractory to standard treatments [3, 4]. Unlike rheumatoid arthritis, development of biologics to treat LN has been challenging, partially owing to its disease heterogeneity [5]. Thus, unmet needs remain for patients with LN who are refractory to conventional remission-induction therapy.

Although abnormalities in acquired immunity, such as the presence of autoreactive T and B cells and autoantibodies, are considered a hallmark of SLE, recent studies have also highlighted the critical roles of innate immunity, including macrophages (Mϕ), in SLE. It has been shown that Mϕ are abundantly present in LN glomerulus and that the number of glomerular Mϕ positively correlates with proteinuria level [6]. Moreover, depletion of Mϕ ameliorated antibody-induced LN in a nephritis model [7]. Recently, the novel concept of Mϕ subsets emerged, comprising proinflammatory, classically activated M1 Mϕ and anti-inflammatory, alternatively activated M2 Mϕ [8]. The balance between M1 and M2 Mϕ has been implicated in the pathogenesis of nephritis [9]. In an adriamycin-induced nephritis model, IL-10/transforming growth factor-β-modified M2 Mϕ adequately protected against renal injury [9]. In renal biopsy specimens from patients with LN, M2c Mϕ (CD68^+^/CD163^+^) predominates over M1 Mϕ (CD68^+^/inducible nitric oxide synthase-positive) [10]. Collectively, these data suggest potential roles of M2 Mϕ in LN.

M2 Mϕ highly express CD163, a scavenger receptor of hemoglobin-derived heme that is widely accepted as one of the surface markers for M2 Mϕ [11, 12]. Also, phosphorylated signal transducer and activator of transcription 1 (pSTAT1) and CMAF have been proposed as M1 and M2 Mϕ markers [13]. Haptoglobin-bound heme captured by CD163 is engulfed into Mϕ lysosomes and degraded into biliverdin and Fe^2+^ by inducible enzyme heme oxygenase (HO)-1. Among the leukocytes, HO-1 is expressed mainly in monocytes/Mϕ lineage cells [14]. Expression of HO-1 is tightly controlled by the transcriptional balance between activator nuclear factor erythroid 2-related factor 2 (Nrf2) and repressor BTB and CNC homology 1 (Bach1) [15, 16]. We and others have previously shown that HO-1 has anti-inflammatory effects and that its induction is beneficial for the treatment of various inflammatory animal models [17]. We previously reported that peritoneal injection of hemin, a chemical inducer of HO-1, into lupus-prone MRL/*lpr* mice suppressed proteinuria and kidney injury [18]. In line with our findings, Nrf2-deficient mice developed lupus-like autoimmune nephritis [19], whereas treatment with Nrf2 activator dimethyl fumarate ameliorated pristane-induced LN [20]. These results reinforce the notion that induction of HO-1 could be beneficial for the treatment of LN. However, it is still unclear whether M2 Mϕ play a pathological role in human LN or whether induction of HO-1 is useful for the treatment of patients with LN.

In the present study, we demonstrate that M2-like Mϕ lacking HO-1 expression are found in LN kidneys. Supplementation of HO-1 by targeting Bach1 genes ameliorated LN in mice, suggesting that dysregulated HO-1^low^ M2 Mϕ contribute to augmenting the inflammation of LN.

## Methods

### Patients

All of the patients fulfilled the revised 1997 American College of Rheumatology criteria for the classification of systemic lupus erythematosus [21]. Patients enrolled in the study signed a written informed consent form that was approved by ethics committee of Yokohama City University Hospital (B130905030).

### Mice

MRL/MpJ JmsSlc^-*lpr*/*lpr*^ (MRL/*lpr*) mice were obtained from Japan SLC (Hamamatsu, Japan). The *Bach1*^*−/−*^ mice (on the C57BL/6J background) used in this study have been described previously [22]. We obtained congenic mice by backcrossing with *Bach1*^−/−^ C57/BL6J for 12 generations. Male *Bach1*^+/−^ and female *Bach1*^+/+^ MRL/*lpr* mice were interbred. Mice were genotyped by PCR using primers previously described [23]. *Bach1*^−/−^ MRL/*lpr* female mice and *Bach1*^+/+^ MRL/*lpr* female mice were used in this study. Animals were maintained under specific pathogen-free conditions within the animal facility at Yokohama City University. Animal treatment protocols were approved by the Yokohama City University animal protocol ethics committee.

Urine was collected for 6 h from individual 24-week-old mice in metabolic cages (Shinano Manufacturing Co., Tokyo, Japan). Urine protein and creatinine concentrations were determined by using DC Protein Assay Reagent (Bio-Rad Laboratories, Hercules, CA, USA) and the Parameter Creatinine assay kit (R&D Systems, Minneapolis, MN, USA). Sera were collected from the tails of 20-week-old mice. Serum anti-double-stranded DNA (anti-dsDNA) antibody (immunoglobulin G [IgG]) and blood urea nitrogen (BUN) were measured using an enzyme-linked immunosorbent assay (Shibayagi, Shibukawa, Japan) [18] and a BUN colorimetric detection kit (Arbor Assays, Ann Arbor, MI, USA).

Mϕ from MRL/*lpr* mice were collected by peritoneal lavage with ice-cold PBS. These cells underwent positive selection by using a CD11b^+^ MACS antibody (Miltenyi Biotec, Bergisch Gladbach, Germany), followed by incubation at 37 °C for 40 minutes to remove floating cells [24].

### *In vitro* polarization of human M1- and M2-like Mϕ

Human peripheral blood mononuclear cells were obtained from heparinized peripheral blood by gradient density centrifugation using Ficoll-Paque medium. Monocytes were purified by nonmonocyte depletion with antibody-conjugated magnetic-activated cell sorting microbeads (MACS II Monocyte Isolation Kit; Miltenyi Biotec). Cells were cultured in RPMI 1640 medium (Sigma-Aldrich, St. Louis, MO, USA) supplemented with 10% FBS (2917354; MP Biomedicals, Santa Ana, CA, USA), 50 mg/ml streptomycin, and 50 U/ml penicillin. To differentiate cells into M2- or M1-like Mϕ, 50 ng/ml macrophage colony-stimulating factor (M-CSF) (216-MC; R&D Systems) or 20 ng/ml granulocyte-macrophage colony-stimulating factor (GM-CSF) (215-GM; R&D Systems) was added, respectively [25]. Cells were incubated for 10 consecutive days, and medium change was performed at days 2, 5, and 8. For some experiments, M1- and M2-like Mϕ were stimulated with lipopolysaccharide (LPS) (1 μg/ml, serotype 0111:B4; InvivoGen, San Diego, CA, USA), interferon (IFN)-α2b (1 U/μl; PBL Assay Science, Piscataway, NJ, USA), or IFN-β (1 U/μl; PBL Assay Science).

### Immunohistochemical analysis

Paraffinized renal biopsy specimens were preserved at Yokohama City University Hospital. Renal pathological findings were evaluated by the pathologists in accordance with the International Society of Nephrology/Renal Pathology Society (ISN/RPS) 2003 classification of LN [26]. Renal biopsy specimens were sectioned at 3-μm thickness, deparaffinized in xylene, hydrated in ethanol, and pretreated with citrate buffer (10 mM sodium citrate, pH 6.0). Slides were autoclaved for 20 minutes, followed by a 30-minute incubation at room temperature. Endogenous peroxidase activity was disrupted with 0.3% H_2_O_2_ in methanol for 30 minutes. Immunohistochemistry was performed on consecutive sections using the following antibodies (Abs): CD68 (PGM1) (1:100 dilution, M0876; Dako/Agilent Technologies, Santa Clara, CA, USA), CD163 (1:200 dilution, UKNDL-L-CD163; Novocastra Laboratories, Newcastle, UK), HO-1 (1:500 dilution, ADI-OSA-110; Enzo Biochem, Farmingdale, NY, USA), and pSTAT1 (1:400 dilution, D3B7; Cell Signaling Technology, Danvers, MA, USA), and CMAF (1:50 dilution, M-153, Santa Cruz Biotechnology, Dallas, TX, USA). Abs were applied for 60 minutes at 25 °C. Slides were developed using the Dako EnVision kit (Agilent Technologies). After hematoxylin staining, total numbers of CD68^+^, CD163^+^, pSTAT1^+^, CMAF^+^, and HO-1^+^ cells within the glomeruli and representative ex-glomerular lesions were counted. The number of M1-like Mϕ and M2-like Mϕ were calculated using the following formula: < CD163 × pSTAT1/(pSTAT1 + CMAF) > and < CD163 × CMAF/(pSTAT1 + CMAF) >, where “CD163 × pSTAT1” refers to cells that are double expressors of CD163 and pSTAT1 and “CD163 × CMAF” refers to cells that are double expressors of CD163 and CMAF. For immunocytochemistry, cells incubated on chamber slides were fixed with 4% paraformaldehyde for 10 minutes, followed by application of the Abs and protocols used in the aforementioned immunohistochemical analysis. In immunofluorescence staining, blocking was performed for 1 h by 1% Tris-buffered saline (TBS)-4% goat serum-TBS. Staining was achieved using primary Ab (CD163, pSTAT1, and CMAF) for 45 minutes at room temperature as described above. We used Alexa Fluor 555 antimouse IgG (A21425, dilution 1:500; Thermo Fisher Scientific, Waltham, MA, USA) and Alexa Fluor 488 antirabbit IgG (A11034, dilution 1:500; Thermo Fisher Scientific) as a second antibody.

### RT-PCR

Total RNA was extracted using the RNeasy Mini Kit (Qiagen, Hilden, Germany), and complementary DNA was prepared with Invitrogen SuperScript II enzyme (Life Technologies, Carlsbad, CA, USA), according to the manufacturers’ protocols. Primers and probes for human and mouse *Hmox1* (Hs00157965_m1, Mm00516004_m1), *CD163* (Hs00174705_m1, Mm00474091_m1), *Bach1* (Hs00230917_m1), *IL-6* (Hs00174131_m1), *IL-10* (Hs00961622_m1), and *Gapdh* (4326317E, Mm99999915_g1) for RT-PCR were purchased from Applied Biosystems (Foster City, CA, USA). Primers for *Ifnα* and *hypoxanthine phosphoribosyltransferase (Hprt)* are listed in Additional file 1: Table S1. RT-PCR was performed using TaqMan Fast Advanced Master Mix (Applied Biosystems) or SYBR Green (Fast SYBR Green Master Mix; Applied Biosystems), and the data were analyzed with the StepOnePlus Real-Time PCR System (Applied Biosystems). The data were standardized to the expression of *Gapdh* or *Hprt*. The comparative cycle threshold method was used to semiquantify messenger RNA (mRNA) levels.

### Western blot analysis

Kidney and spleen samples were lysed in lysis buffer (150 mM NaCl, 50 mM 4-[2-hydroxyethyl]-1-piperazineethanesulfonic acid, 1 mM ethylenediaminetetraacetic acid, 1% Triton X-100) in the presence of a protease inhibitor (Roche, Mannheim, Germany). After 10-minute incubation, cell membranes were disrupted by ultrasonication (Emerson Electric, St. Louis, MO, USA). Supernatants were collected after centrifugation at 15,000 rpm for 30 minutes and adjusted to an appropriate concentration with LDS sample buffer (Life Technologies) and 2-mercaptoethanol (Sigma-Aldrich). Each lysate was resolved by NuPAGE 4–12% Bis-Tris gel electrophoresis (Life Technologies) and transferred onto polyvinylidene fluoride (PVDF) membrane (Merck Millipore, Darmstadt, Germany). After blocking with 5% skim milk PBS, the PVDF membrane was probed with antimouse HO-1 monoclonal antibody (1:500 dilution) or antimouse Bach1 monoclonal antibody (1:100 dilution, F-9; Santa Cruz Biotechnology) overnight at 4 °C, followed by incubation with ECL antimouse IgG horseradish peroxidase-linked whole antibody (1:5000 dilution; GE Healthcare Life Sciences, Little Chalfont, UK) for 60 minutes at room temperature. Blots were developed using ECL Prime Western Blotting Detection Reagent (GE Healthcare Life Sciences) and exposed to the LAS-3000 Mini Imaging System (FUJIFILM, Tokyo, Japan) for 1–5 minutes.

### Statistical analysis

Statistical analysis was performed by using Prism software (GraphPad Software, La Jolla, CA, USA). Data are presented as mean and SEM. *p* < 0.05 was considered statistically significant.

## Results

### Characteristics of M2-like Mϕ in glomeruli of patients with LN

In this study, we enrolled 19 patients with LN, including 13 patients with LN newly diagnosed via renal biopsy (Table 1). The histopathological diagnosis of LN was made by light microscopy and a routine set of immunofluorescence studies. According to ISN/RPS classification, 18 of the 19 patients were categorized into class III or class IV, and pure class V was found in the remaining patient (Table 1). Five patients with LN classes I and II were also evaluated.

**Table 1:** Clinical features of patients with lupus nephritis at renal biopsy

Characteristics of all patients with SLE (*n* = 19)	Data
Female sex, *n* (%)	15 (78.9)
Age, years	31.0 ± 10.9
Duration of SLE, years	6.1 ± 7.6
Duration of LN, years	2.8 ± 5.8
Initial diagnosis of LN at renal biopsy, *n* (%)	13 (68.4)
ISN/RPS classification, *n*	
III (A)	4
(A/C)	6 (3)^a^
IV-S (A)	0
(A/C)	3 (1)^a^
-G (A)	2
(A/C)	3
V	1
SLEDAI score	16.0 ± 8.9
PSL, *n* (%)	12 (63.2)
Dosage of PSL, mg/day	15.0 ± 12.4
Concomitant immunosuppressants^b^, *n* (%)	4 (21.1)
Urine protein, g/24 h	1.88 ± 1.93
Serum C3, mg/dl	60.1 ± 22.7 (normal range 70–129)
Serum C4, mg/dl	10.6 ± 6.9 (normal range 12–36)
Serum creatinine, mg/dl	0.65 ± 0.18 (normal range 0.48–0.82)
Anti-DNA- or anti-dsDNA antibody-positive^c^, *n* (%)	17 (89.4)

*Abbreviations: dsDNA* Double-stranded DNA, *ISN/RPS* International Society of Nephrology/Renal Pathology Society, *LN* Lupus nephritis, *SLE* Systemic lupus erythematosus, *SLEDAI* SLE Disease Activity Index, *PSL* Prednisolone, *C3* Complement component C3, *C4* Complement component C4

Data are shown as number (%) or mean ± SD

^a^Numbers in parentheses show number of patients with class V component

^b^Concomitant immunosuppressants included mycophenolate mofetil, cyclophosphamide, azathioprine, and tacrolimus

^c^Anti-DNA and anti-dsDNA antibody were determined by radioimmunoassay and enzyme-linked immunosorbent assay, respectively

**Fig. 1:**
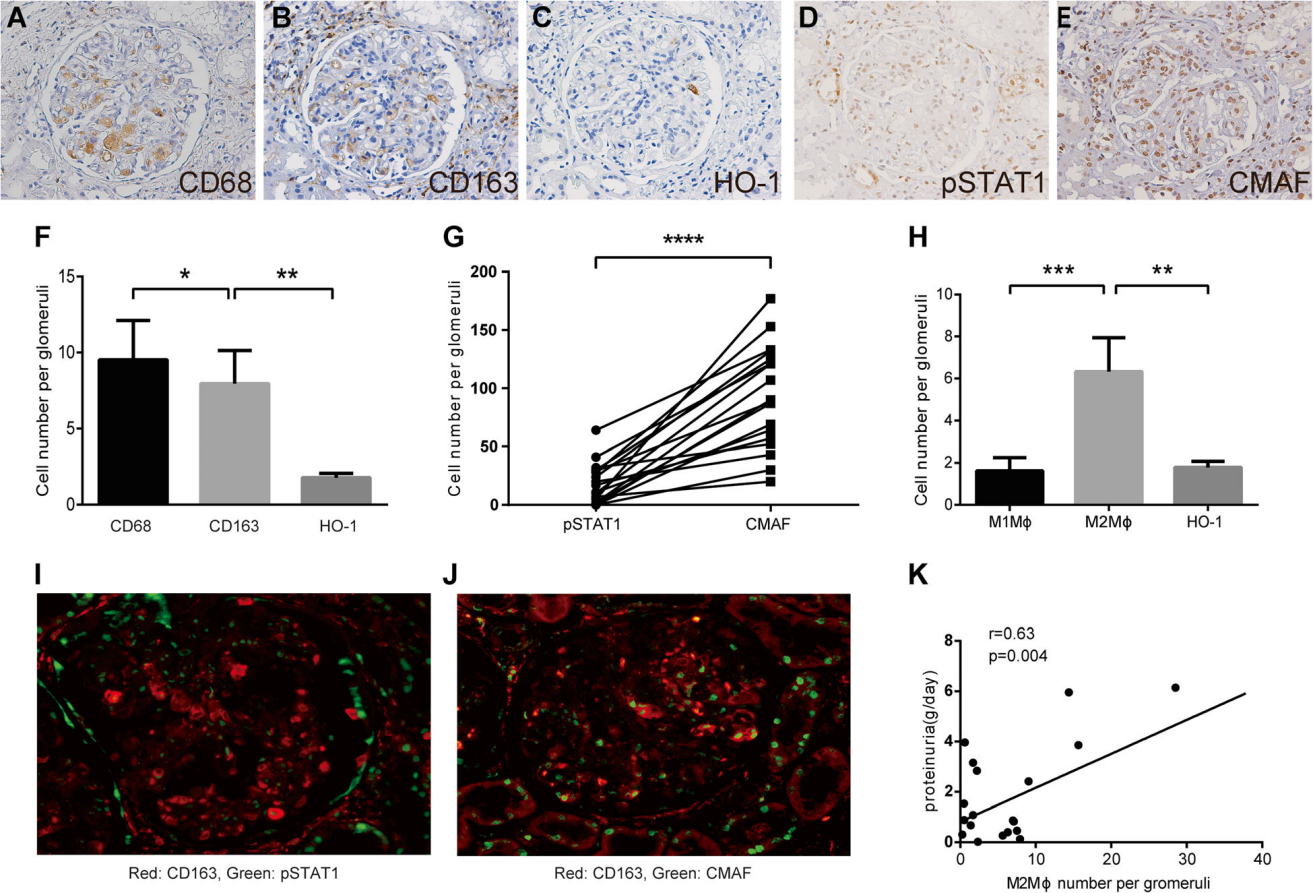
Numbers of CD68^+^, CD163^+^, and heme oxygenase (HO)-1^+^ cells in the glomerulus of patients with lupus nephritis. Representative immunohistochemical images of the renal biopsy specimen obtained from a patient with lupus nephritis (International Society of Nephrology/Renal Pathology Society [ISN/RPS] class IV-G [A/C]) are shown. Serial sections of a glomerulus stained with antibodies against (a) CD68, (b) CD163, (c) HO-1, (d) phosphorylated signal transducer and activator of transcription 1 (pSTAT1), and (e) CMAF (original magnification × 400). f Numbers of CD68^+^, CD163^+^, and HO-1^+^ cells in glomerulus of renal tissue from patients with systemic lupus erythematosus (SLE) (*n* = 19) were counted. Data shown are mean ± SEM. g Numbers of pSTAT1^+^ and CMAF^+^ cells in a glomerulus of renal tissue from patients with SLE (*n* = 19) were counted. h Numbers of estimated M1 macrophage (Mϕ), M2 Mϕ, and HO-1^+^ cells in glomerulus in the renal tissues from patients with SLE (*n* = 19). Data shown are mean ± SEM. i and j Representative immunofluorescently stained images of the renal biopsy specimen from a patient with lupus nephritis (ISN/RPS class IV-G [A/C]). *Red* shows CD163; *green* shows pSTAT1 (i) or CMAF (j). Double-positive cells indicate M1-like Mϕ and M2-like Mϕ in (i) and (j), respectively. k Correlation between proteinuria levels (g/day) and numbers of M2-like Mϕ within the glomeruli (*n* = 19). *p < 0.05, **p < 0.01, ***p < 0.001, ****p < 0.0001 by Student’s *t* test

Expression of CD68, CD163, HO-1, pSTAT1, CMAF, and HO-1 was determined in the renal biopsy specimens using immunohistochemistry. Representative immunohistochemical data from a patient with LN (ISN/RPS class IV-G [A/C]) are shown in Fig. 1a–e. Average cell numbers of CD68, CD163, and HO-1 per glomerulus in the renal tissues from the patients with LN were 9.52, 7.95, and 1.78, respectively (Fig. 1f). We found that numbers of CD163^+^ cells were comparable to numbers of CD68^+^ cells in the glomerulus of LN. We also found that the number of CMAF^+^ M2-like cells was greater than that of pSTAT1^+^ M1-like cells in the glomerulus of LN (Fig. 1g). Average cell numbers of M1-like Mϕ, M2-like Mϕ, and HO-1 per glomerulus of patients with LN were 1.62, 6.33, and 1.78, respectively (Fig. 1h). Double-immunofluorescence staining in a representative case indicated that CD163^+^CMAF^+^ M2-like Mϕ are dominant compared with CD163^+^pSTAT1^+^ cells in the glomeruli of LN (Fig. 1i, j). Numbers of M2-like Mϕ per glomeruli positively correlated with urine protein levels (Fig. 1k), consistent with a previous report [6]. The number of Mϕ in glomerulus were few in LN classes I and II (Additional file 1: Figure S1). In extraglomerular lesions, of LN classes III, IV, and V, the number of M2-like Mϕ was greater than M1-like Mϕ. Also, HO-1^+^ cells were significantly fewer than numbers of M2-like Mϕ (Additional file 1: Figure S2).

Previous reports have shown that HO-1 mediates the anti-inflammatory property of M2 Mϕ [27–29]. Nevertheless, the numbers of HO-1^+^ cells were fewer than those of M2-like Mϕ. These data suggest that HO-1 expression is downregulated in glomerular Mϕ of human LN in spite of the M2-like property.

### HO-1 expression is high but is repressed by type I interferons in human M2-like Mϕ generated *in vitro*

HO-1 is an inducible protein, and the expression is positively and negatively modulated by external environments [27]. We hypothesized that local inflammatory conditions of LN are responsible for the discrepancy between CD163/CMAF positivity and HO-1 expression in glomerular Mϕ. In the present study, we focused on effects of IFN-α on mRNA expression of both molecules in human monocyte-derived M2-like Mϕ generated *in vitro*, because type I IFN and the related genes are upregulated in patients with LN, including the renal tissues as a so-called IFN signature [1, 3, 30].

**Fig. 2:**
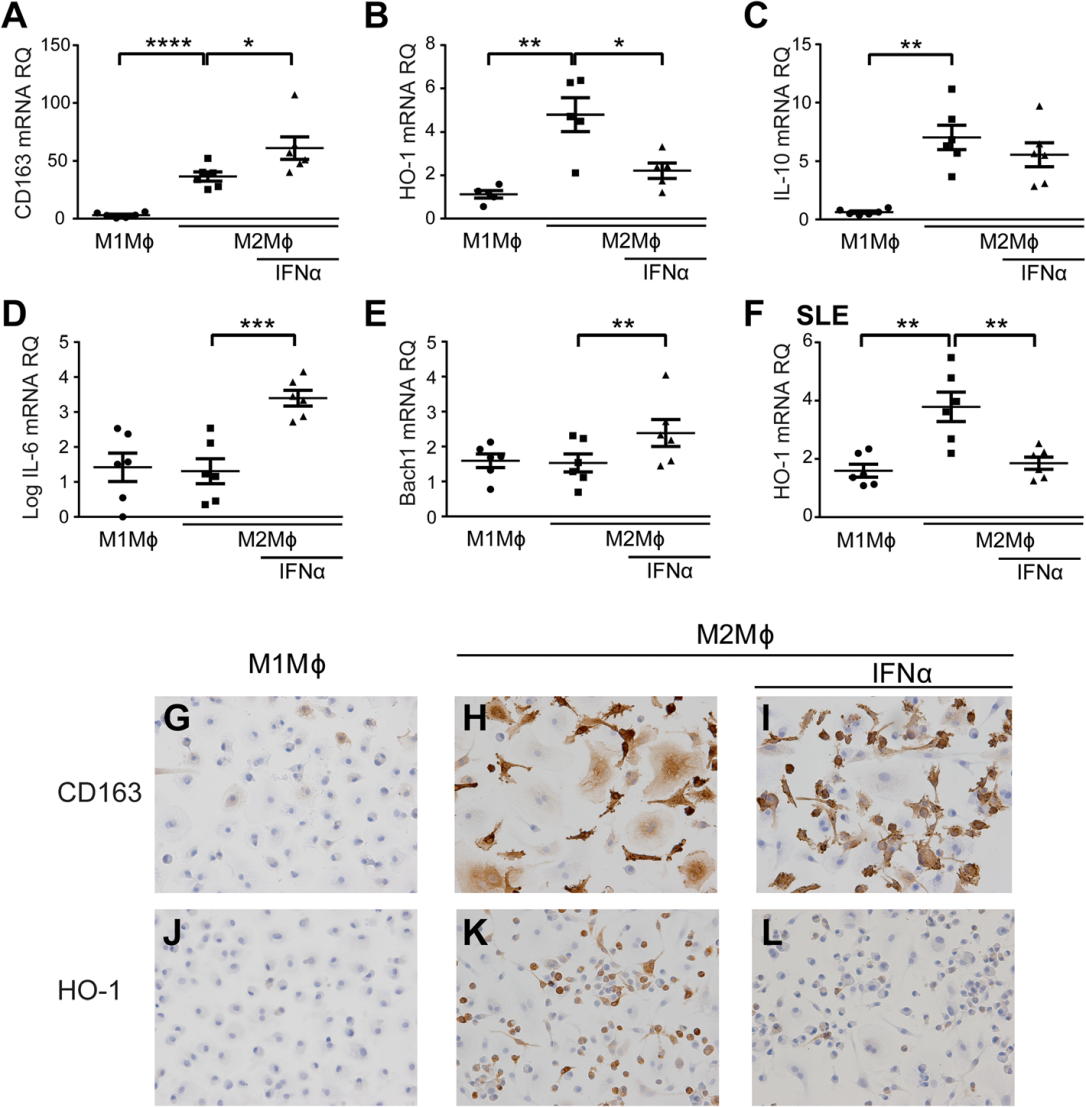
Expression of CD163, heme oxygenase (HO-1), and BTB and CNC homology 1 (Bach1) in human monocyte-derived M1- and M2-like macrophages (Mϕ) from healthy donors (HD). Purified peripheral monocytes were cultured in the presence of granulocyte-macrophage colony-stimulating factor or macrophage colony-stimulating factor for 10 days to induce differentiation of M1- or M2-like Mϕ, respectively. Messenger RNA (mRNA) expression was semiquantitatively determined from M1- and M2-like Mϕ generated from HD *in vitro* (*n* = 6) by RT-PCR of (a) CD163, (b) HO-1, (c) interleukin (IL)-10, (d) IL-6, and (e) Bach1. f HO-1 mRNA expression of M1- and M2-like Mϕ from patients with systemic lupus erythematosus (SLE) stimulated with interferon (IFN)-α (*n* = 6). *p < 0.05, **p < 0.01, ***p < 0.001, ****p < 0.0001 by Student’s *t* test. Data shown are mean ± SEM. Representative immunocytochemical staining shows results for M1- and M2-like Mϕ stimulated with or without human recombinant IFN-α. CD163 (g–i) and HO-1 (j–*l*) (original magnification × 400). *RQ* Relative quantification

Concordant with previous reports [11, 12, 28], our results confirmed that both CD163 and HO-1 mRNA and protein expression was high in M2-like Mϕ compared with M1-like Mϕ in our *in vitro* culture systems (Fig. 2a, b, g–*l*). The *in vitro* M2-like polarization was associated with increased mRNA of IL-10, another hallmark of M2 Mϕ, whereas that of IL-6 was comparable between M1- and M2-like Mϕ (Fig. 2c, d). The established M2-like Mϕ were further treated with type I IFNs for 4 h. The results showed that HO-1 expression was suppressed by IFN-α in M2-like Mϕ (Fig. 2b, k, and *l*) [14, 31], whereas mRNA of Bach1, an HO-1 transcriptional repressor, was upregulated (Fig. 2e). Simultaneously, IL-6 mRNA levels, but not IL-10 levels, were upregulated by IFN-α. The findings were also found in M2-like Mϕ treated with either IFN-β or LPS (Additional file 1: Figure S3A). Moreover, the same results were reproduced in M2-like Mϕ derived from patients with SLE (Fig. 2f). The results suggest that the type I IFN-enriched condition in the glomerular lesions of LN suppresses HO-1 expression through upregulated Bach1 and modulates anti-inflammatory properties in M2-like Mϕ.

### HO-1 upregulation by genetic ablation of Bach1 ameliorates LN in mice

We and others have reported that induction of HO-1 expression is beneficial in the treatment of LN [18, 19]. Our *in vitro* experiments suggest the involvement of Bach1 in IFN-α-dependent reduction of HO-1 expression in LN. To determine the role of Bach1 in the development of LN, we generated LN-prone MRL/*lpr* mice lacking Bach1 and asked whether recovery of HO-1 expression in M2 Mϕ by Bach1 deficiency leads to amelioration of LN.

**Fig. 3:**
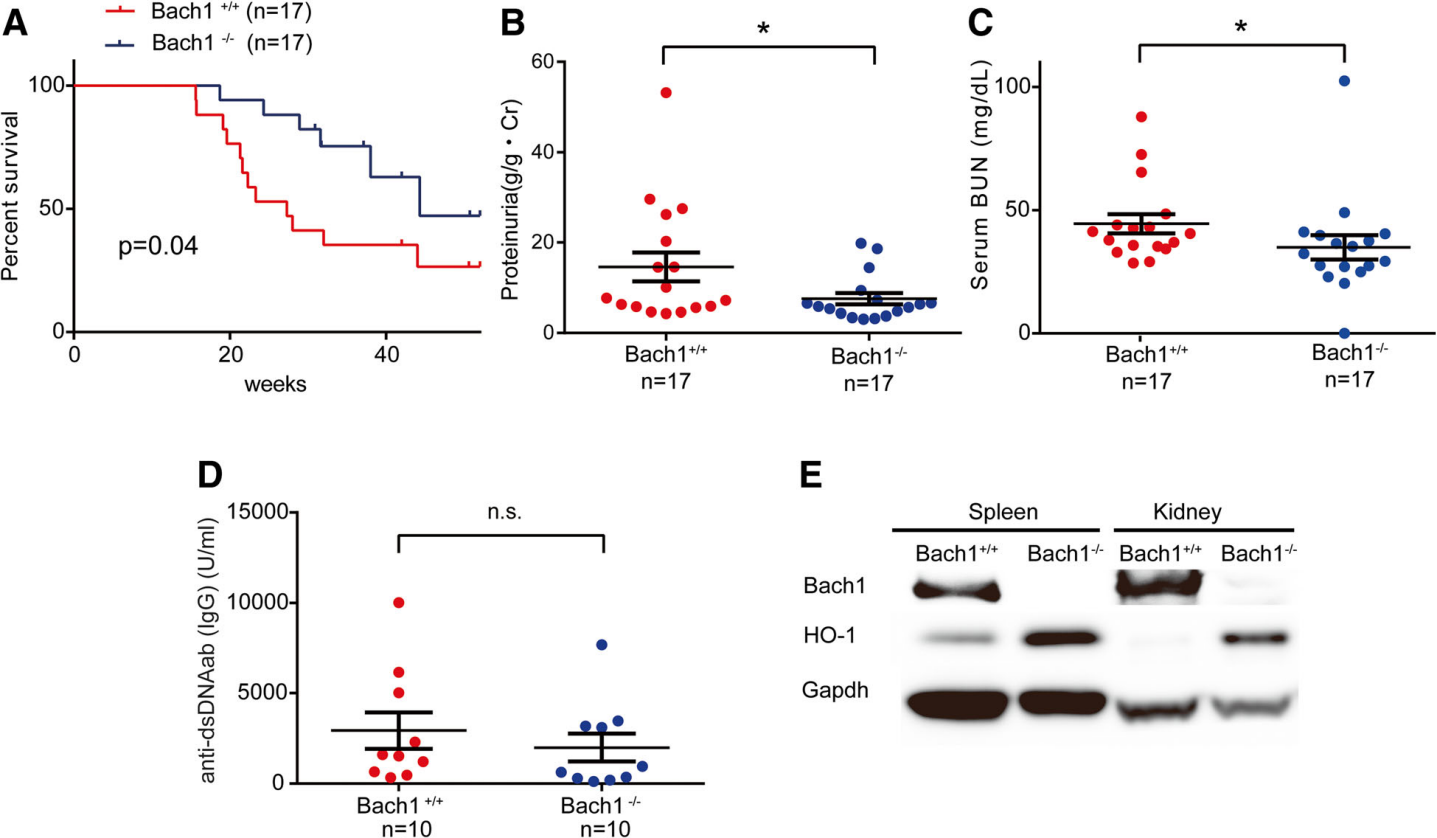
Phenotypic analysis of BTB and CNC homology 1 (Bach1)-deficient MRL/*lpr* mice. Clinical and renal findings were compared between *Bach1*^+/+^ and *Bach1*^−/−^ MRL/*lpr* mice as follows. a Kaplan-Meier survival analysis. b Proteinuria (g/g*Cr). c Serum blood urea nitrogen (BUN) (mg/dl) at 24 weeks. d Anti-double-stranded DNA (anti-dsDNA) antibody (Ab) immunoglobulin G (IgG) (U/ml). *p < 0.05 by Student’s *t* test. *n.s.* Not significant. Data shown are mean ± SEM. e Immunoblot analysis of Bach1, heme oxygenase (HO)-1, and glyceraldehyde 3-phosphate dehydrogenase (Gapdh) in whole spleens and kidneys

**Fig. 4:**
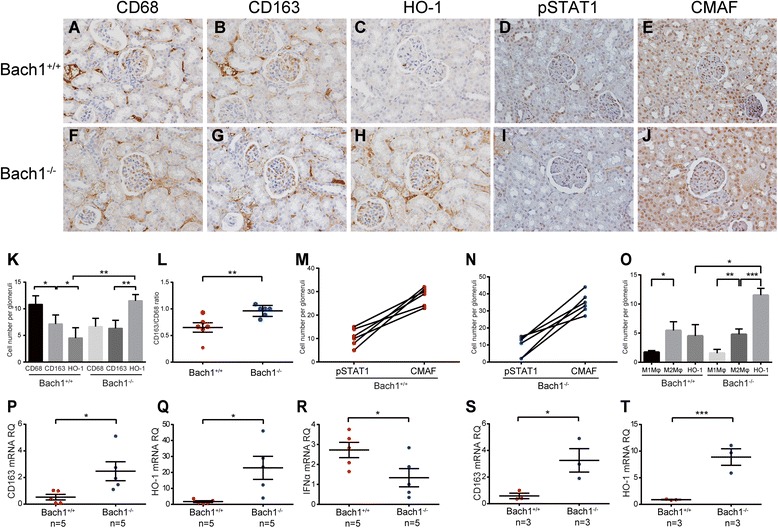
Characteristics of cells within the glomeruli of BTB and CNC homology 1 (Bach1)-deficient MRL/*lpr* mice. Immunohistochemical staining of the renal tissues for CD68 (a, f), CD163 (b, g), heme oxygenase (HO)-1 (c, h), phosphorylated signal transducer and activator of transcription 1 (pSTAT1) (d, i), and CMAF (e, j) (original magnification × 400). k Numbers of CD68^+^, CD163^+^, and HO-1^+^ cells in glomeruli of mice. *l* Ratio of CD163^+^ and CD68^+^ cells within the glomerulus of *Bach1*^+/+^ and *Bach1*^−/−^ MRL/*lpr* mice. m and n Numbers of pSTAT1^+^ and CMAF^+^ cells in glomeruli of *Bach1*^+/+^ and *Bach1*^−/−^ MRL/*lpr* mice (*n* = 2 from each group). o Numbers of estimated M1 macrophage (Mϕ), M2 Mϕ, and HO-1^+^ cells in glomeruli in the renal tissues from *Bach1*^+/+^ and *Bach1*^−/−^ MRL/*lpr* mice. Data shown are mean ± SEM. Three glomeruli from each mouse were evaluated. (p) CD163, (q) HO-1, and (r) interferon (IFN)-α messenger RNA (mRNA) from the whole kidneys. mRNA expression of (s) CD163 and (t) HO-1 of CD11b^+^ peritoneal Mϕ. *p < 0.05, **p < 0.01, ***p < 0.001 by Student’s *t* test. Data shown are mean ± SEM

Microsatellite analysis (ICLAS Monitoring Center, Kawasaki, Japan) confirmed the genetic purity of *Bach1*^−/−^ MRL/MpJ JmsSlc^-*lpr*/*lpr*^ was > 99.4% compared with wild type (Additional file 1: Figure S4). PCR analysis confirmed *Bach1* deficiency in these congenic mice (Additional file 1: Figure S5). Using female mice, we observed their phenotypic changes, including renal function, over the time course. As shown in Fig. 3a, the survival rate of *Bach1*^−/−^ MRL/*lpr* mice was better than that of *Bach1*^+/+^ MRL/*lpr* mice. Urine total protein/creatinine ratio at 24 weeks and serum BUN at 20 weeks were decreased in *Bach1*^−/−^ mice compared with *Bach1*^+/+^ mice (Fig. 3b, c), though there was no difference in anti-dsDNA Ab(IgG) between the mice (Fig. 3d). The discrepancy between proteinuria/serum BUN and the serological findings suggest that *Bach1* gene targeting modulates the innate immune process after immune complex (IC) deposition, resulting in favorable clinical outcomes of *Bach1*^−/−^ MRL/*lpr* mice. Therefore, we further focused on the analysis of the renal lesions (Fig. 4a–j).

Immunoblot analysis confirmed the deficiency of Bach1 and increased expression of the HO-1 protein in the kidneys and spleens of *Bach1*^−/−^ compared with *Bach1*^+/+^ MRL/*lpr* mice (Fig. 3e). Concordantly, immunohistochemical analysis revealed more HO-1-expressing cells in glomeruli from *Bach1*^−/−^ mice than in those from *Bach1*^+/+^ MRL/*lpr* mice, whereas no differences were found in CD68- or CD163-expressing cells (Fig. 4c, h, and k). However, M2-like polarization was more prominent in glomerular Mϕ of *Bach1*^−/−^ MRL/*lpr* mice than in the wild-type mice, as shown by the increased ratio of CD163^+^ cells per CD68^+^ cells (Fig. 4*l*). CMAF^+^ cells were much more numerous than pSTAT1^+^ cells in both *Bach1*^*+/+*^ and *Bach1*^*−/−*^ mice (Fig. 4m, n). The estimated number of M2-like Mϕ was greater than M1 Mϕ in both *Bach1*^+/+^ and *Bach1*^−/−^ mice. However, numbers of HO-1^+^ cells in glomeruli were greater in *Bach1*^*−/−*^ mice (Fig. 4o). PCR analysis also revealed higher CD163 and HO-1 mRNA expression in the kidneys of *Bach1*^−/−^ mice than in wild-type mice, whereas the IFN-α expression level was rather decreased in *Bach1*^−/−^ MRL/*lpr* mice (Fig. 4p–r).

To further compare the characteristics of Mϕ in these mice, Mϕ obtained from peritoneal lavage were examined (Fig. 4s, t). We found that Mϕ from *Bach1*^−/−^ MRL/*lpr* mice expressed higher levels of CD163 and HO-1 mRNA than wild-type mice did, suggesting that M2-like polarization is more evident in Bach1 deficiency, consistent with the results of a previous paper [24]. Collectively, these data suggest that Bach1 negatively regulates HO-1 expression and M2 Mϕ polarization, leading to the inflammation of LN.

## Discussion

In the present study, we showed high numbers of M2-like Mϕ than M1-like Mϕ within the glomerulus of patients with LN, suggesting an M2 shift in LN. However, HO-1 expression was reduced in these abundantly present M2-like Mϕ (Fig. 1). Moreover, human monocyte-derived M2-like Mϕ treated with type I IFNs showed reduced HO-1 and increased Bach1 expression. Mϕ from Bach1-deficient mice showed an M2 shift along with high HO-1 expression, consistent with a previous report [24]. These data suggest that HO-1-expressing M2 Mϕ are necessary to regulate LN.

Over the past few decades, authors of hundreds of papers have reported the anti-inflammatory properties of HO-1 in various inflammation settings. Indeed, HO-1 deficiency in humans and mice exhibits marked inflammation caused by oxidative stress [32, 33]. Moreover, we previously reported that HO-1 knockdown by small interfering RNA resulted in an enhanced inflammatory response in human monocytes [14]. Therefore, it is likely that these “HO-1-deficient M2-like Mϕ” in LN are proinflammatory rather than anti-inflammatory as in ordinary M2 Mϕ. Indeed, the results of the present study show low HO-1 and high IL-6 production in human M2 Mϕ stimulated with IFN-α (Fig. 2b and d). Besides, a previous study demonstrated that chemical induction of HO-1 was beneficial in the LN murine model [18]. Along the same line, the present study provides hope that the cell population could be reversed into anti-inflammatory M2 Mϕ through induction of HO-1 via inhibition of Bach1.

**Fig. 5:**
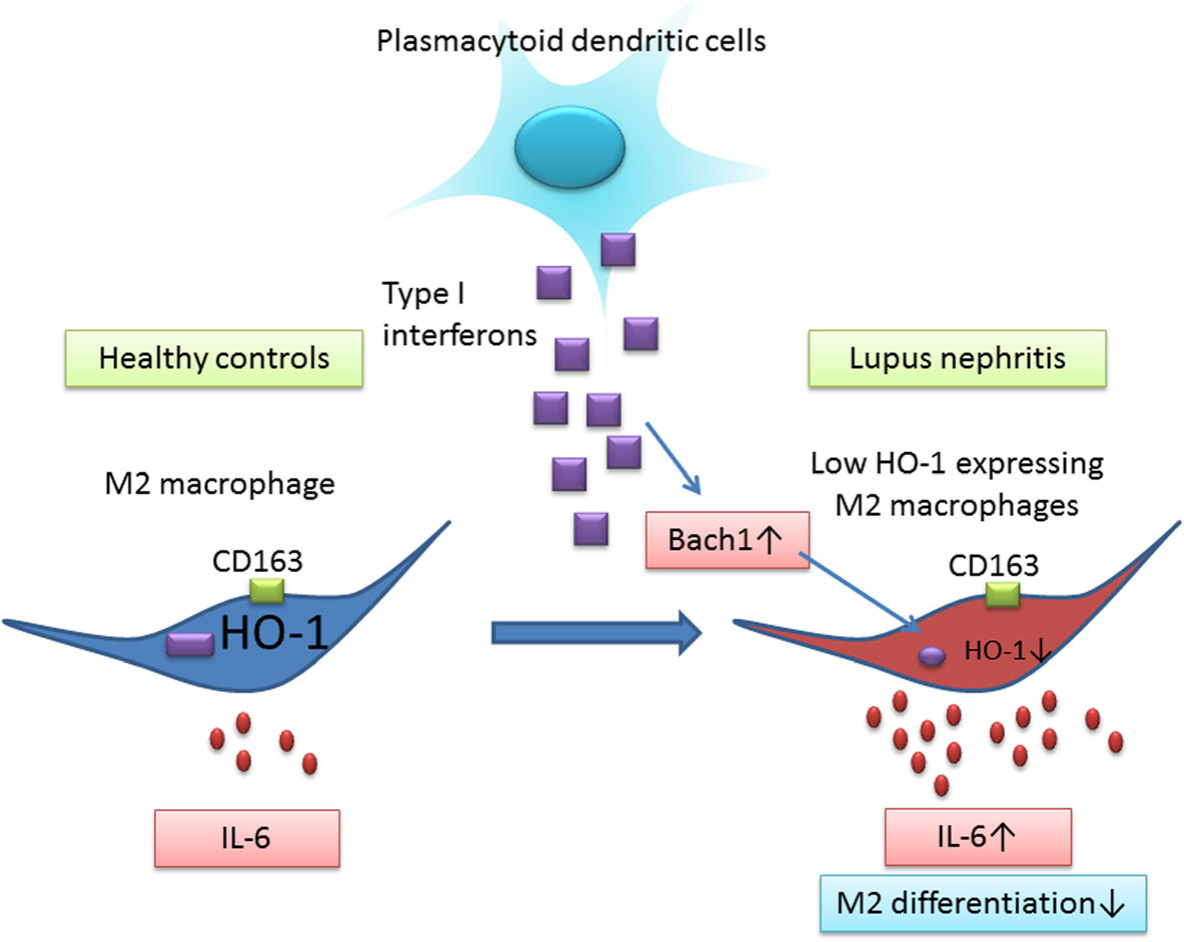
Schema describing our hypothesis of BTB and CNC homology 1 (Bach1)-mediated M2 dysregulation in lupus nephritis. Type I interferons are highly produced by plasmacytoid dendritic cells in systemic lupus erythematosus [1]. The secreted interferons suppress heme oxygenase (HO)-1 expression in CD163^+^ M2-like macrophages via induction of HO-1 transcriptional repressor Bach1. These aberrantly functional HO-1 low M2-like macrophages produce inflammatory cytokines such as interleukin (IL)-6, which is facilitated by reduced HO-1 expression. Bach1 also has the property of modulating M2-like macrophage differentiation, as suggested in our *Bach1*^−/−^ mouse experiments

LN is considered a representative IC disease caused by anti-dsDNA Ab IC deposition in the glomerulus. Subsequently, the deposited IC triggers activation of the complement system and preferentially promotes M2 Mϕ polarization [34]. Our data show that anti-inflammatory properties of glomerular M2 Mϕ are abrogated by a systemic and local IFN signature that suppresses HO-1 through upregulation of Bach1. Moreover, costimulation with Toll-like receptor ligands, which reduced HO-1 expression in this study, and IgG promotes inflammatory responses of M2 Mϕ instead of suppressing inflammation via Fcγ receptor IIa [35]. Our *in vitro* analysis suggests that the functional changes of M2-like Mϕ are associated with an altered cytokine profile characterized by increased IL-6 synthesis while maintaining IL-10 production capacity (Fig. 2). Both cytokines are implicated in the pathogenesis of SLE, because recent genetic studies have identified the *IL10* risk allele in SLE [36]. Our hypothesis proposed on the basis of this study is summarized in Fig. 5. Collectively, M2 Mϕ dysfunction may facilitate the inflammation in LN. Bach1-mediated HO-1 supplementation is one of the promising strategies to “tame” these aberrantly activated M2 Mϕ in LN.

In the present study, we demonstrate that Bach1 is induced by type I IFNs in human M2-like Mϕ (Fig. 2e). Elucidating the mechanisms of how type I IFNs regulate Bach1 expression is essential. Bach1 was initially identified as a Maf-binding protein, and it now is well established that Bach1 forms a heterodimer with small Maf [37]. A recent study showed that MafB is involved in the regulation of IRF3-dependent type I IFN-inducible genes [38]. Interestingly, genome-wide association studies (GWAS) identified Bach2, which belongs to the same family as Bach1, as being associated with SLE [39]. These findings and our data suggest that Bach1 is critically involved in type I IFN-mediated inflammation, including SLE.

Bach2 regulates antibody class switch in B cells [40], thus contributing to acquired immunity because it is identified as a susceptible gene in GWAS of various autoimmune diseases [39]. In contrast, a primary source of Bach1 is monocytes (BioGPS GeneAtlas U133A, gcrma), and *Bach1* was identified as one of the core Mϕ-associated genes in mice [41], suggesting that Bach1 plays a strong role in innate immunity. In this context, Bach1-deficient mice showed a milder LN phenotype than wild-type mice without affecting anti-dsDNA antibody production (Fig. 3c). This fact suggests that innate immune responses after IC deposition on glomerulus are also significant in this model. Although the mainstay of current treatment strategies for LN is to suppress acquired immunity, including pathogenic autoantibody production, our data support an alternative treatment strategy directed against innate immunity via Bach1. The approach is expected to have an advantage over conventional therapies regarding safety issues, especially for complications with infection because systemic immunosuppressive effects are marginal.

There are several possible strategies to treat LN by targeting the Bach1, HO-1, and M2 Mϕ. First, inhibition of Bach1 transcription is promising. Recently, the novel Bach1 inhibitor HPP971, which increases HO-1 expression, was developed [42]. Nrf2, a competitor to Bach1, is an alternative because Nrf2-deficient mice showed a phenotype resembling LN [43]. Treatment with novel Nrf2 inducer TFM-735 ameliorated experimental autoimmune encephalomyelitis [44]. Finally, supplementation or generation of normal HO-1-expressing M2 Mϕ could also be beneficial for LN. It has been shown that adoptive transplant of M2 Mϕ, but not of M1 Mϕ, reduced SLE severity in clodronate- and activated lymphocyte-derived DNA-treated mice [45].

## Conclusions

Our data suggest that functional alteration of M2 Mϕ plays an important role in LN and that Bach1 is a therapeutic target for LN.

## Additional file

Additional file 1: **Table S1.** Primer sequences used for qRT-PCR. **Figure S1.** Numbers of CD68, CD163, HO-1 positive cells in the glomerulus of lupus nephritis patients (ClassI or II). **Figure S2.** Numbers of CD68, CD163, HO-1 positive cells in the extra-glomerulus of lupus nephritis patients. **Figure S3.** HO-1 mRNA expression in M1 and M2 Mϕ stimulated with various regents. **Figure S4.** Genomic background of congenic mice. **Figure S5.** Genotyping of Bach1 knockout mice. (DOCX 953 kb)

## Abbreviations

Ab: Antibody
Bach1: BTB and CNC homology 1
BUN: Blood urea nitrogen
C3: Complement component C3
C4: Complement component C4
dsDNA: Double-stranded DNA
*Gapdh*: Glyceraldehyde 3-phosphate dehydrogenase
GM-CSF: Granulocyte-macrophage colony-stimulating factor
GWAS: Genome-wide association studies
HD: Healthy donors
HO-1: Heme oxygenase 1
*Hprt*: Hypoxanthine phosphoribosyltransferase
IC: Immune complex
IFN: Interferon
IgG: Immunoglobulin G
IL: Interleukin
ISN/RPS: International Society of Nephrology/Renal Pathology Society
LN: Lupus nephritis
LPS: Lipopolysaccharide
M-CSF: Macrophage colony-stimulating factor
mRNA: Messenger RNA
Mϕ: Macrophage
Nrf2: Nuclear factor erythroid 2-related factor 2
PSL: Prednisolone
pSTAT1: Phosphorylated signal transducer and activator of transcription 1
PVDF: Polyvinylidene fluoride
RQ: Relative quantification
SLE: Systemic lupus erythematosus
SLEDAI: Systemic Lupus Erythematosus Disease Activity Index
TBS: Tris-buffered saline
