# Supplementary material for: Dysregulated heme oxygenase-1low M2-like macrophages augment lupus nephritis via Bach1 induced by type I interferons
Source: Arthritis Res Ther. 2018 Apr 10;20:64. doi: 10.1186/s13075-018-1568-1 (PMC5894134; doi:10.1186/s13075-018-1568-1)
Supplement: Supplementary file 1 — Table S1. Primer sequences used for qRT-PCR. Figure S1. Numbers of CD68, CD163, HO-1 positive cells in the glomerulus of lupus nephritis patients (ClassI or II). Figure S2. Numbers of CD68, CD163, HO-1 positive cells in the extra-glomerulus of lupus nephritis patients. Figure S3. HO-1 mRNA expression in M1 and M2 Mϕ stimulated with various regents. Figure S4. Genomic background of congenic mice. Figure S5. Genotyping of Bach1 knockout mice. (DOCX 953 kb) [file 13075_2018_1568_MOESM1_ESM.docx]

**Supplementary materials for: Dysregulated HO-1-low M2-like macrophages augment lupus nephritis via Bach1 induced by type-I interferons**

Daiga Kishimoto^1^, Yohei Kirino^1^, Maasa Tamura^1^, Mitsuhiro Takeno^3^, Yosuke Kunishita^1^, Kaoru Takase-Minegishi^1^, Hiroto Nakano^1^, Ikuma Kato^2^, Kiyotaka Nagahama^4^, Ryusuke Yoshimi^1^, Kazuhiko Igarashi^5^, Ichiro Aoki^2^, and Hideaki Nakajima^1^

^1^ Yokohama City University Graduate School of Medicine, Department of Stem Cell and Immune Regulation, Yokohama, Japan

^2^ Yokohama City University Graduate School of Medicine, Department of Molecular Pathology, Yokohama, Japan

^3^ Nippon Medical School Graduate School of Medicine, Department of Allergy and Rheumatology, Tokyo, Japan

^4^ Kyorin University School of Medicine, Department of Pathology, Tokyo, Japan

^5^ Tohoku University Graduate School of Medicine, Department of Chemistry, Sendai, Japan

**Supplementary figure 1. Numbers of CD68, CD163, HO-1 positive cells in the glomerulus of lupus nephritis patients (ClassI or II)**

**
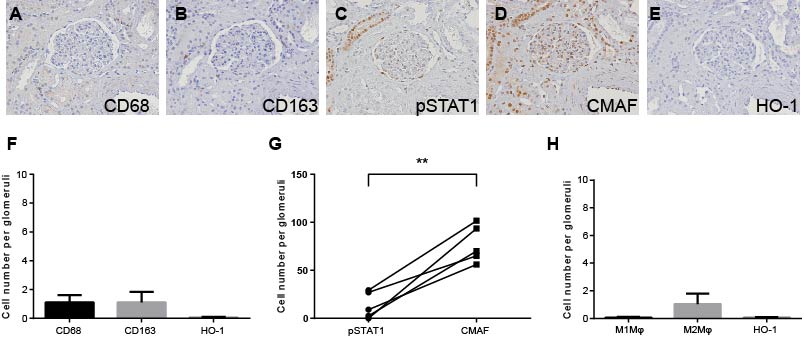
**

Representative immunohistochemical images of the renal biopsy specimen from a lupus nephritis patient (ISN/RPS class II). Serial sections of a glomerulus stained with antibodies against A; CD68, B; CD163, C; pSTAT1, D; CMAF, and E; HO-1 (x400). F; Numbers of CD68, CD163, HO-1 positive cells in a glomerulus were counted in the renal tissues from SLE patients (n=5). Data were shown as mean + SEM. G; Numbers of pSTAT1 and CMAF positive cells in a glomerulus were counted in the renal tissues from SLE patients (n=5). H; Numbers of estimated M1 Mφ, M2 Mφ and HO-1 positive cells in a glomerulus in the renal tissues from SLE patients (n=5). Data were shown as mean + SEM. **p<0.01 by student *t*-test.

**Supplementary figure 2. Numbers of CD68, CD163, HO-1 positive cells in the extra-glomerulus of lupus nephritis patients**

**
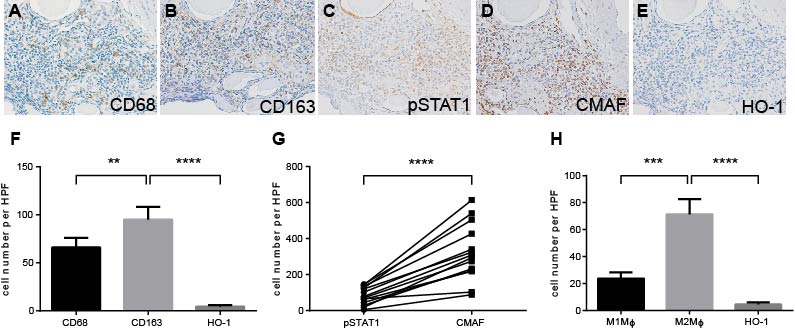
**

Representative immunohistochemical images of the renal biopsy specimen from a lupus nephritis patient (ISN/RPS class IV-G(A/C)). Serial sections of an extra-glomerulus lesion stained with antibodies against A; CD68, B; CD163, C; HO-1, D; pSTAT1, and E; CMAF (x400). F; Numbers of CD68, CD163, HO-1 positive cells in an extra-glomerulus were counted in the renal tissues from SLE patients (n=19). Data were shown as mean + SEM. G; Numbers of pSTAT1 and CMAF positive cells in an extra-glomerulus were counted in the renal tissues from SLE patients (n=19). H; Numbers of estimated M1 Mφ, M2 Mφ and HO-1 positive cells in an extra-glomerulus in the renal tissues from SLE patients (n=19). Data were shown as mean + SEM. **p<0.01, ***p<0.001, ****p<0.0001 by student *t*-test.

**Supplementary figure 3. HO-1 mRNA expression in M1 and M2 Mϕ stimulated with various regents.**

**
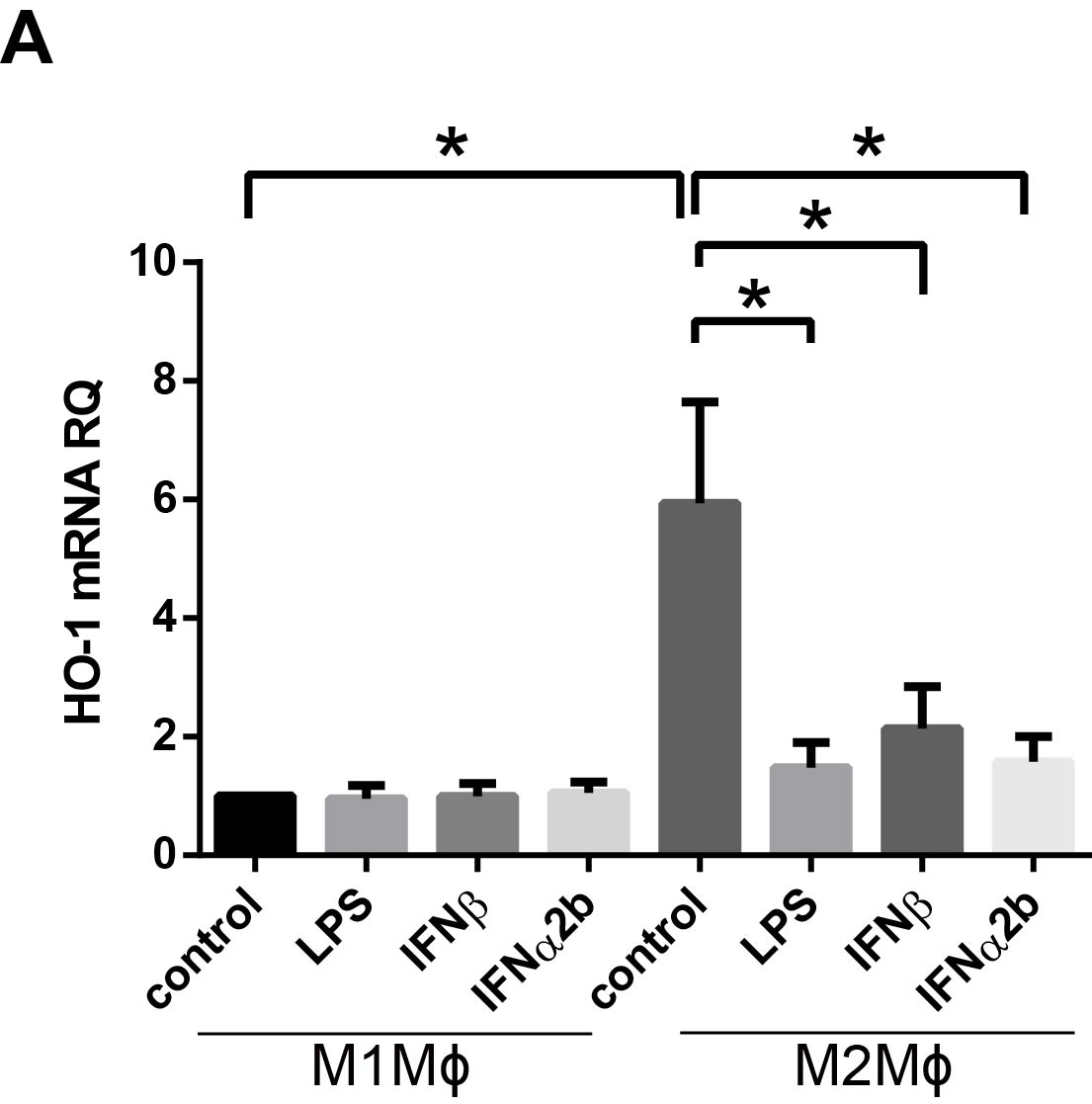
**

HO-1 mRNA expressions of M1 and M2 Mϕ from HC stimulated with either LPS, IFNβ, or IFNα2b (n=4). *p<0.05, **p<0.01 by student *t*-test. Data are shown as mean + SEM.

**Supplementary figure 4. Genomic background of congenic mice.**

Informative 85 short tandem repeat markers were used to compare C57BL/6J (B6), N12, and MRL/lpr strain (MRL). Note that Chr. 16 25.77cM is located near *Bach1*locus.

**Supplementary figure 5. Genotyping of Bach1 knockout mice.**

PCR of Bach1 gene with genomic DNA was performed to evaluate Bach1 deficiency in mice.

**Supplementary Table 1. Primer sequences used for qRT-PCR.**

| Target |  | Sequence(5’-3’) | Ref |
| --- | --- | --- | --- |
| *Ifnα(non-α4)* | Fwd | ARSYTGTSTGATGCARCAGGT | [47] |
|  | Rev | GGWACACAGTGATCCTGTGG |  |
| *Hprt* | Fwd | GATTAGCGATGATGAACCAGGTT |  |
|  | Rev | CCTCCCATCTCCTTCATGACA |  |
